# Computational Methods for Strain-Level Microbial Detection in Colony and Metagenome Sequencing Data

**DOI:** 10.3389/fmicb.2020.01925

**Published:** 2020-08-18

**Authors:** Christine Anyansi, Timothy J. Straub, Abigail L. Manson, Ashlee M. Earl, Thomas Abeel

**Affiliations:** ^1^Delft Bioinformatics Lab, Delft University of Technology, Delft, Netherlands; ^2^Infectious Disease and Microbiome Program, Broad Institute of MIT and Harvard, Cambridge, MA, United States; ^3^Department of Immunology and Infectious Diseases, Harvard T.H. Chan School of Public Health, Boston, MA, United States

**Keywords:** metagenomics, microbial detection, strain-level classification, methods review, whole genome sequencing, bioinformatics

## Abstract

Metagenomic sequencing is a powerful tool for examining the diversity and complexity of microbial communities. Most widely used tools for taxonomic profiling of metagenomic sequence data allow for a species-level overview of the composition. However, individual strains within a species can differ greatly in key genotypic and phenotypic characteristics, such as drug resistance, virulence and growth rate. Therefore, the ability to resolve microbial communities down to the level of individual strains within a species is critical to interpreting metagenomic data for clinical and environmental applications, where identifying a particular strain, or tracking a particular strain across a set of samples, can help aid in clinical diagnosis and treatment, or in characterizing yet unstudied strains across novel environmental locations. Recently published approaches have begun to tackle the problem of resolving strains within a particular species in metagenomic samples. In this review, we present an overview of these new algorithms and their uses, including methods based on assembly reconstruction and methods operating with or without a reference database. While existing metagenomic analysis methods show reasonable performance at the species and higher taxonomic levels, identifying closely related strains within a species presents a bigger challenge, due to the diversity of databases, genetic relatedness, and goals when conducting these analyses. Selection of which metagenomic tool to employ for a specific application should be performed on a case-by case basis as these tools have strengths and weaknesses that affect their performance on specific tasks. A comprehensive benchmark across different use case scenarios is vital to validate performance of these tools on microbial samples. Because strain-level metagenomic analysis is still in its infancy, development of more fine-grained, high-resolution algorithms will continue to be in demand for the future.

## Introduction

Within a species, bacteria can be highly diverse in terms of their virulence, resistance to antibiotics, geographical transmission patterns, and other phenotypic characteristics (Fournier et al., 2014; Maxson and Mitchell, 2016). Individual strains can vary greatly with respect to pathogenicity, treatment options, transmissibility, and growth rate (Balmer and Tanner, 2011; Alizon et al., 2013). In order to effectively treat patients, study bacterial population dynamics, conduct epidemiological surveillance, and stem outbreaks, it is critical to identify which specific strains of a species present in a sample (Fournier et al., 2014; Deurenberg et al., 2017). Tracking and comparing individual strains shared across sets of samples would allow for the assessment of the evolution of population diversity in longitudinal samples within a patient or other host system. The ability to identify specific strains in a noisy background of other organisms present in a metagenomic sample could allow for improved tracking of strains involved in an outbreak across a population.

Accurately identifying specific pathogenic strains would aid in patient diagnosis, allowing for personalized treatment regimens, improved treatment outcomes, and a reduction in the spread of antibiotic resistance. Mixed infections, defined as infections caused by multiple strains of a single pathogen species (Marshall, 2002; Cohen et al., 2012), represent an underappreciated challenge to understanding infections and have been described for at least 22 bacterial species (Balmer and Tanner, 2011), including *M. tuberculosis* (Cohen et al., 2012; Plazzotta et al., 2015), *C. difficile* (Eyre et al., 2013, 2012), and *Streptococcus pneumoniae* (Esposito et al., 2002; Minagawa et al., 2008). It is estimated that 10–20% of *M. tuberculosis* patients in high risk areas (Huang et al., 2010; Navarro et al., 2011; Plazzotta et al., 2015) and 10% of *Staphylococcus aureus* (Lessing et al., 1995; Cespedes et al., 2005) patients are infected with multiple pathogenic strains. Mixed infections put patients at a higher risk of treatment failure (Balmer and Tanner, 2011; Cohen et al., 2012; Plazzotta et al., 2015), as strains with different drug susceptibility and antibiotic resistance profiles (Falagas et al., 2008; El-Halfawy and Valvano, 2015) can complicate diagnosis and identification of the optimal treatment regimen (Balmer and Tanner, 2011). In addition to poor treatment outcomes, mixed strain infections can increase pathogen virulence due to selective pressure within the host (Frank, 1996). Accurate classification of individual strains is critical for identifying mixed infections and will help determine proper treatment options for patients with complex infections, track transmission of pathogenic strains in a population, and differentiate between reinfection and intra-host pathogen evolution.

While there is clearly substantial value in being able to pinpoint individual strains within metagenomic samples, most current widely used tools for metagenomic analysis only allow for an assessment of composition at the genus or species level, not the strain level. For example, the current most popular metagenomics taxonomic classification programs, including Kraken (Wood et al., 2014) MetaPhlAn2 (Truong et al., 2015) and GTDB-Tk (Chaumeil et al., 2019), are capable of identifying mixed populations only at the species or genus level–not at the individual strain level within a species. Tools capable of conducting classification of metagenomic samples for higher taxonomic levels such as the family, genus, or species have been previously reviewed (Hunter et al., 2012; Mande et al., 2012; Teeling and Glöckner, 2012; Goldman and Domschke, 2014). In contrast, tools to detect taxonomy at a finer-grained taxonomic levels within metagenomic samples – targeting specific strains within a species – are still in their infancy (Marx, 2016; Segata, 2018), with most tools only published within the past 5 years.

To date, there have been no reviews focused on strategies to computationally classify heterogeneous bacterial populations using WGS data at the level of specific strains within a species. This literature review gives an overview of recent methods for classification at the intra-species, or strain level, including methods based on WGS data to identify both specific strains, as well as mixes of strains. These tools are divided into assembly based, alignment based, and reference free methods. We have included both secondary sources (reviews or methods papers) and original research, where the main objective is developing a novel methodology for detecting heterogeneous bacterial communities, e.g., mixed infections or within host evolution. The majority of these tools operate using short-read sequencing data, due to the abundance and affordability of the Illumina platform. However, the advent of both long-read sequencing and single-cell sequencing holds great promise in enabling effective strain-level identification. We also cover the few presently existing metagenomic tools specifically made for these sequencing platforms in this review. Although we focus on clinical applications here, the methods discussed are applicable to a broad range of biological ecosystems typically analyzed using metagenomics, including soil, wastewater or other environments. We discuss appropriate applications of each strategy, evaluation of these strategies in literature, as well as the applicability of these algorithms to health and disease.

## Approaches for Detecting Individual Strains of Bacteria Within a Species

Currently available approaches to classifying genetically distinct populations from a sequencing read set can be binned into three categories (see Table 1): (i) methods using (metagenomic) assembly or *de novo* reconstruction of genomes within the sample (assembly based), (ii) aligning genomes to a reference database (including full genome alignment based and pattern based), and (iii) reference database free approaches that rely on applying statistics directly to allele (variant) frequencies.

**TABLE 1:** Tool benchmark and technical details.

Author	Method name	Type^1^	Technical details^2^	Sample benchmarks^3^	Test metrics^4^	Required coverage level per strain^5^
Pulido-Tamayo et al., 2015	EVORha	assembly based	– java	– *E. coli* time series (lab grown) – *C. difficile* mixed infection samples	reliability score, mean absolute error, rmse	50× coverage
Quince et al., 2017	DESMAN	assembly based	– git/python – linear runtime – 5 strains in 117 min	– fecal metagenome samples – community of 100 species and 210 strains with 96 samples (synthetic)	accuracy	–
Ahn et al., 2015	Sigma	alignment based	– C++ – scaled for supercomputers (alignment with 10,000 cores takes 10 min) – sample with 5 strains takes 20 h and 62GB RAM on a computer with 64CPU	– fecal metagenome dataset – numerous spike ins of fecal set to simulate outbreaks	accuracy, TP/FP	0.02× coverage
Sankar et al., 2015	BIB	alignment based	– 1 million reads in 10 min on single CPU – git/python	– mixtures of 2–6 staphylococcus strains (synthetic) – *S. aureas* sample data	absolute error	
Francis et al., 2013; Byrd et al., 2014; Hong et al., 2014	Pathoscope	alignment based	– git/BioConda – 1 sample using 16 CPU and 256GB RAM took 17 min	– European *E. coli* outbreak 2011 (O104:H4) – mixed read datasets of 3 strains	TP/FP	20% genome coverage
Fischer et al., 2017	DiTASiC	alignment based	– git/conda – requires R and python	– 3 simulated set groups – low, medium, and high complexity metagenomic benchmark datasets (synthetic) – lacks real world testing	sum of squared errors, TP/FP/FN/FP –	–
Huson et al., 2007	MEGAN	alignment based	– gui/java – took 180 h using 64CPU for 300 k reads	– Sargasso sea dataset – mammoth bone – simulation studies – mostly species level testing	FP	–
Dilthey et al., 2019	MetaMaps	alignment based	– git/Perl – takes 16–210 h using 262GB RAM – cannot make own DB	– simulated data – human microbiome project data (PacBio, species) – Zymo synthetic community (Oxford Nanopore Technology)	Precision, recall	–
Smillie et al., 2018	StrainFinder	pattern based^6^	– git/python – 100 samples across 649 reference genomes using 100–200cores takes 48 + hours – needs alignment file with some preprocessing as input	– 2–32 strains across 2–64 samples (synthetic) – recurrent *C. difficile* infection over time	Unifrac distance	25×
Gan et al., 2016		pattern based^6^	– not available	– TB datasets	–	1× coverage
Luo et al., 2015	ConStrains	pattern based^6^	– git/python – took 8.5 h and 2 GB ram on infant gut dataset – custom DB not possible	– *E. coli* admixtures 2–7 strains (synthetic) – gut microbiome time series – microbiome time series (synthetic) – cystic fibrosis patient infection data	Jenson-Shannon divergence	10× coverage
Freitas et al., 2015	GOTTCHA	pattern based^6^	– git/Perl – used 16cores and 132GB RAM while being 2–5× slower than other tools – custom DB not possible	– human microbiome project mixtures of 22 genomes – spiked air filter metagenome spiked – spiked human stool – synthetic communities of 25–300 genomes	precision, recall, F-score, false discovery rate and accuracy	
Sahl et al., 2015	WG-FAST	pattern based^6^	– conda – uses phylogeny	– fecal specimens *E. coli* O104:H4 outbreak	accuracy	1×
Roosaare et al., 2016	StrainSeeker	pattern based^6^	– online web tool – Perl/R – needs 300GB space to build DB – uses 1 cpu, 512GB RAM and took 1.1 min for classification	– *E. coli*, *K. pneumoniae*, *E. faceilius*, *S. enterica* isolate identification (synthetic)	accuracy	<1× coverage
Albanese and Donati, 2017	StrainEst	pattern based^6^	– git/docker/python – takes 12–25 min for a 10× –100× coverage sample using 129–591MB RAM and 4 cores	– paired strains from 4 species (synthetic) – 2 HMP mock communities (21 organisms) – specific strain in skin microbiome – cross sectional *E. coli* strains in stool samples – gut microbiome time series	Matthew Correlation Coefficient, Jensen-Shannon divergence	10× coverage
Truong et al., 2017	StrainPhlAn	pattern based^6^	– git/conda	– human microbiome	accuracy	2×
Nayfach et al., 2016	MIDAS	pattern based^6^	– git/docker/python – on 1CPU process 5,000 reads per second using 3 GB RAM – 1.5–2 h for typical gut metagenome	– stool metagenomes time series – marine metagenomes	(only of genes) accuracy, TP/FP	1 × coverage
Costea et al., 2017	metaSVN	pattern based^6^	– git/conda – 676 samples in 223 min using 2,488 GB RAM and 32 cores	– oral metagenome	–	5 × coverage
Tu et al., 2014	GSMer	pattern based^6^	– git/Perl scripts	– diabetes patients gut microbiome – obesity associated microbiome	TP	<0.25 × (100 GSMs) >0.25 × (50 GSMs)
Scholz et al., 2016	PanPhlAn	pattern based^6^	– git/python	– *E. coli* outbreak O104:H4 – gut microbiomes – skin microbiome – oral microbiome – marine metagenomes	F1 score	1 × coverage
Koslicki and Falush, 2016	MetaPalette	pattern based^6^	– git/docker/python	– spiked HMP community (22 organisms) – soil metagenome	Divergence, FP	22 × coverage
Anyansi et al., 2020	QuantTB	pattern based^6^	– git/python – <10 min for single sample using single core and pre-build database	– TB datasets	precision, recall, F-score, FP/TP	–
Eyre et al., 2013		reference db free	– R script in supplements	– *C. difficile* infected patients	RMSE	–
O’Brien et al., 2016	pfmix	reference db free	– R – for a 5 strain sample takes 10 min on single core	– blood from malaria patients	Mean squared error	25 reads
Assefa et al., 2014	*estMOI*	reference db free	– git/Perl – little documentation	– clinical isolates of *P. falciparum*	accuracy	30 × coverage
Zhu et al., 2017	DEploid	reference db free	– R package – 1–6 h	– clinical isolates *of P. falciparum*	accuracy	1% abundance
Sobkowiak et al., 2018	MixInfect	reference db free	– R script/git – no documentation	– tested on TB samples	accuracy	10 × coverage

*^1^Category of algorithm. ^2^Details about the computational parameters of the tool in terms of code base/runtime/memory usage/availability. ^3^Example datasets tool was tested on in paper. ^4^Metrics by which each method was evaluated. ^5^The required coverage for the tool per stain to perform. If no value is indicated, this indicates the particular value could not be determined from the article where the method was published. ^6^Pattern based methods use a database of predefined markers to classify genetic diversity within a sample.*

### Assembly Based Approaches for *de novo* Strain Level Reconstruction

**FIGURE 1:**
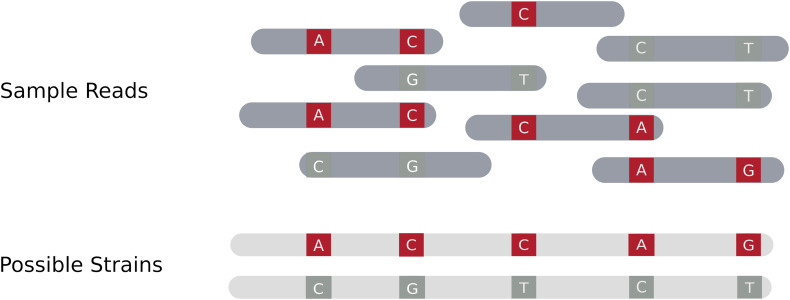
Assembly of multiple distinct strains from a read set. The blue areas in the sample reads represent regions where the strains have identical sequence. Variant locations in the reads are denoted as red or dark gray stripes. Red variants originate from one haplotype, whereas dark gray variants originate from the other. The goal of an assembly based method is to resolve distinct strains based on the coverage and distribution of the read data, drawing on methods previously developed for resolving haplotypes.

Assembly based approaches attempt to identify individual strains in a mixture by performing (whole) genome assembly, drawing on tools developed for haplotype (single clone or strain) reconstruction in diploid species. To obtain an accurate reconstruction there must be a sufficient number of sites that differ between the component strains in order to separate or cluster variants into distinct strains (Yuan et al., 2012; Votintseva et al., 2017). Therefore, accurate reconstruction of distinct strains requires sufficient read length to capture overlap between reads, enough discriminating sites to separate populations, and the presence of at least one variant site in most reads. Figure 1 gives an overview of how a read set can be resolved into a set of distinct individual strains using an assembly based procedure.

EVORhA, one of the few assembly based methods designed for reconstructing complete bacterial genomes from bulk metagenomic sequencing data, identifies strains via local haplotype assembly (Table 1; Pulido-Tamayo et al., 2015). For each genomic region containing a sufficient amount of genetic variation, candidate strains are first defined as individual genetically distinct combinations of polymorphisms. To filter out candidate strains that are actually sequencing errors, a minimum number of reads must support an initial candidate strain. In an extension step, candidates are merged with nearby locally constructed candidate strains, based on read frequency and overlap of polymorphism combinations. Ultimately, a mixture model is used to group extended candidate strains occurring at similar frequencies and match these together on a genome-wide level, making the read frequency ratios of observed candidate strains crucial to this method. However, this read frequency criteria for merging strains can produce chimeric strains due to the presence of subpopulations with similar frequencies, similar to a key problem encountered in phasing with whole genome assembly. Given very high coverage, sufficient frequency diversity and sufficient segregating sites, assembly based methods such as EVORhA can resolve the full genomes of genetically distinct subpopulations and yield the most accurate strain identification results when compared to other categories of strain-level identification tools.

Knowing the full sequences of organisms within a sample then allows for comparison and tracking of strains at the highest resolution possible. As such, these methods would be suitable for observing a strain’s evolutionary trajectory as well as detecting mixed infections composed of strains that are highly similar to each other. In order to estimate frequencies, a method would need to account for relative abundance of reads specific to each strain. DESMAN (Quince et al., 2017) does this by exploiting differences in read coverage between genes conserved within a species and other parts of the genome. DESMAN requires a group of metagenome assembled genomes (MAGS) to do estimate relative abundances.

A major drawback of assembly based methods is that a large amount of coverage, 50–100 × for each strain, is required to achieve an accurate reconstruction, demanding extremely high depth sequencing for strains at a low abundance within a sample (Zagordi et al., 2011). High levels of coverage are required to account for errors introduced by sequencing: each distinct strain must be sequenced with sufficient coverage in order to differentiate spurious variation from true distinct strains. Such high coverages can be achieved in studies where sample complexity is low, with typically less than 5 strains present.

### Reference Database Approaches

In order to relate strains observed within a sample to previously studied genomes or species, it is necessary to use a reference database. Reference databases can vary greatly in different dimensions, such as genome quantity or species diversity. Methods employing a reference database can be broken down into two major categories: (i) approaches that have full genomes within their database, and (ii) approaches that only use subsets of these genomes within their database. Here we cover these two overarching approaches and show the pros and cons of each.

## Full Genome Alignment Based Approaches

**FIGURE 2:**
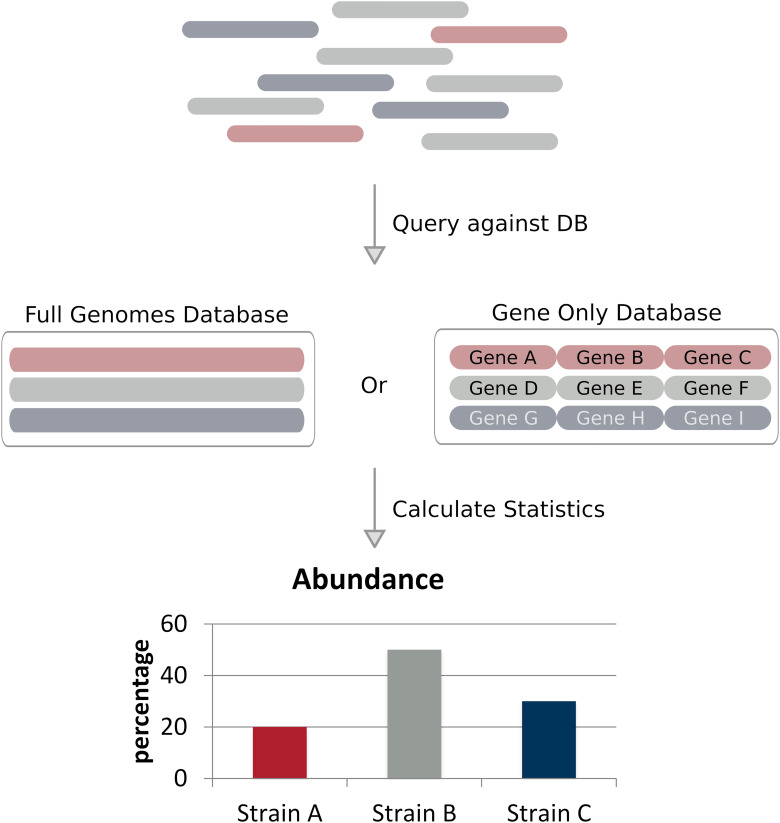
Alignment based approaches. Reads of a sequencing dataset – where different colors denote genetically distinct strains – are aligned to a reference database of full genomes or taxonomic markers (in this case genes). Strain abundances can be estimated by the relative number of reads aligning to each reference genome.

Full genome alignment based methods (alignment methods for short) classify strains by aligning reads to a predefined set of reference genomes and applying probabilistic models to calculate a statistical measure representing the likelihood a specific read is associated with a given reference (Figure 2 and Table 1; Li and Homer, 2010). These methods are often considerably faster than assembly based methods and require less coverage, some methods claim to work with less than 1× coverage. These methods can achieve such low coverages compared to assembly based methods due to their use of a reference database – where the most likely candidate is selected based on the available data using the probabilistic model. Alignment based methods share the same similarities and limitations, such as reference database composition, alignment method, and strain abundance quantification. We will discuss these similarities and limitations on the whole toward the end of this section.

Pathoscope, (Hong et al., 2014) one of the most commonly used classification pipelines for metagenomic analysis, uses different aligners three aligners [GNUMAP (Clement et al., 2009), Bowtie 2 (Langmead and Salzberg, 2012) and BLAST (Altschul et al., 1997)] to align reads to reference genomes. Scores for each alignment are converted to posterior probabilities that represent the likelihood that an alignment is the source of the read. Non-unique reads are reassigned to their nearest reference using a Bayesian mixture model which uses both the mapping scores and the proportions of non-unique reads. Another alignment based method, Sigma, allows users to choose their own short-read alignment algorithm, using Bowtie2 as a default (Ahn et al., 2015). Instead of using scores given by an aligner, Sigma computes its own probability scores for each read to originate from an alignment by examining the number of matches and mismatches between the two.

Calculation of strain abundance in alignment based approaches leverages the number of reads mapping to each reference genome. For Sigma the relative abundance of a genome is simply the proportion of aligned reads out of the total number of reads, whereas Pathoscope calculates relative abundance from the sum of the probability of reads mapped to different genomes in the reference database. BIB exploits the similarities between alignment based strain identification and the more well-established field of RNA-seq data analysis (Kim and Salzberg, 2011; Glaus et al., 2012; Langmead and Salzberg, 2012; Trapnell et al., 2012) for calculating relative abundances, by implementing the RNA-seq algorithm BitSeq (Glaus et al., 2012) within its identification pipeline to calculate relative abundances, after aligning reads to a reference database with Bowtie 2. Unlike other alignment methods, StrainFinder (Smillie et al., 2018) calculates abundances for all the genomes in the reference database using SNP frequencies after aligning reads with BWA. Because StrainFinder uses the Expectation Maximization algorithm to estimate strain frequencies, the user needs to input the expected number of strains expected to be in the sample, to ensure the best likelihood. This not only makes StrainFinder exceptionally computationally intensive, but also makes it less suitable for broad metagenomic studies with unknown number of strains.

While alignment based detection methods work well for species with clear and well-separated sub-lineages, the selection of genomes and choice of size for the reference database is critical for applications to more closely related strains. Some tools aim to draw on large and comprehensive databases in order to gain higher resolution. Sigma offers users the opportunity to define their own reference databases and claims support for up to tens of thousands of genomes. The entirety of RefSeq (2266 genomes at time of publication) has been used as the reference database for Sigma. PathoScope generates a reference database from all genome sequences in NCBI for a given query taxID. The resulting redundancy from using a taxID which could potentially include very closely related strains, instead of a database of filtered genomes such as RefSeq, ensures coverage at all genomic levels, but can result in non-specific strain identification calls. Even if similar sequences are excluded, it is often not practical to have a reference genome for every genetically distinct, closely related strain in a species. While a large reference database can increase coverage of intra-species diversity, it also requires a larger computational search space for matching reads. In addition, differentiating between closely related strains in a highly comprehensive reference database is nearly impossible and can result in an inflated number of false positive predictions. Removal of closely related reference genomes when using BIB improved accuracy and reduced non-specific predictions to multiple unrelated strains. Therefore, proper pruning of representative reference sequences to an appropriate level of resolution is essential.

A major drawback of alignment based methods is that they are dependent on details of the underlying alignment tool and its parameters. Different alignment methodologies can result in discordant results between methods and impacts our ability to perform comparisons between tools. For example, most alignment based methods use a short-read aligner (Hong et al., 2014; Ahn et al., 2015; Sankar et al., 2015), while DiTASiC (Fischer et al., 2017) uses the pseudo alignment approach found in Kallisto (Bray et al., 2016) used for aligning RNA seq reads. Some strain identifiers [Pathoscope, and MEGAN (Huson et al., 2007)] make predictions using the quality score of the alignment of each read. Sigma and BIB use Bowtie2 as an aligner by default which reports all reads that map in multiple locations while Pathoscope and DiTASiC (Fischer et al., 2017) post-process multi-mapping reads within their algorithm, and StrainFinder uses BWA which randomly assigns multi-mapping reads to a specific location. Sigma additionally allows users to select their own aligner. The differences between alignment methods and their impact on results have been reported before in literature (Canzar and Salzberg, 2017). Because these strain classification methods depend on the information given via the alignment, variation at the alignment stage may have consequences throughout the entire method. Each approach can limit the ability to correctly identify strains in a sequencing set in different circumstances. The impact of these variations has not yet been characterized, but will ultimately depend on the species under examination and the parameters of the alignment method and how the classification methods employ the alignment information.

## Pattern Based Methods Based on Alignment to Genetic Markers

Methods where alignments are done to a set of genetic marker, rather than complete genomes were developed to offer decreased compute time and memory requirements. We will refer to these as pattern based methods. These methods classify genetic diversity within a sample using a database of predefined markers, such as unique genes, SNPs, genome-specific k-mers, or fluctuations in GC content. The choice of marker type can vary based on the species, data type, and classification goals. Similar to alignment based methods, pattern based identification methods require a reference database with which to “learn” parameters for their statistical models. However, pattern based methods first preprocess the reference database, extract useful features, and apply these features for a new classifier algorithm, resulting in decreased run times. New sequencing reads can then be classified based off the constructed model.

An example of a method that uses a database of universal single-copy gene families as the predefined marker set is MIDAS, which aims to provide both species and strain-level taxonomic identification. MIDAS first determines species content by aligning reads to a single-copy gene database containing a single representative genome per species (Nayfach et al., 2016). In order to determine strain-level information, reads are mapped to a pan-genome database containing genes from the species found in the first alignment step. Abundance estimation per strain is calculated by normalizing by the coverage of universal single copy gene families. However, this sort of strain level inference using variation in genes alone is not practical for discrimination purposes, because universal single-copy genes represent a smaller portion of the genome and are, by definition, conserved between strains of species (Jordan et al., 2002; Martín et al., 2003). MIDAS requires at least 1 × coverage per strain to determine the presence or absence of a gene.

K-mers are often used in pattern based methods because unlike genes, they are sampled across the whole genome, including regions that are not especially conserved. In order to gain greater resolution than can be obtained by using only genes, GSMer identifies strains by capitalizing upon a strain-specific database of strain-specific k-mers, or GSMs (genome specific markers) (Tu et al., 2014). Each strain in the database is represented by a set of at least 50 GSMs (optimized for k-mer size and number). If a strain has fewer than 50 unique GSMs, it is not included in the database. A strain is only identified in a read set if a perfect match for all 50 GSMs of that strain is identified within the read set, resulting in a high false negative rate and an inability to identify strains not similar to those in the database. This may work well for slow evolving and well conserved organisms that will not change and can be expected to always include the set of 50 GSMers required to be identified. But not in settings where strains are diverse and quickly changing as there is a higher chance for the set 50 GSMers required to be present to have been mutated or changed due to evolutionary drift.

Phylogenetic trees complement pattern based methods by offering a more informative database structure where paths can be indexed with a series of markers leading to a presence of a particular strain. Trees also provide an intuitive visualization of the phylogenetic placement of a strain. Given the tree, these tools map k-mers or SNPs from unknown samples onto nodes within the tree to determine phylogenetic “paths,” sequences of nodes, which represent presence of a particular strain in the sample. Strain abundances are calculated based on the SNP or k-mer coverage.

**FIGURE 3:**
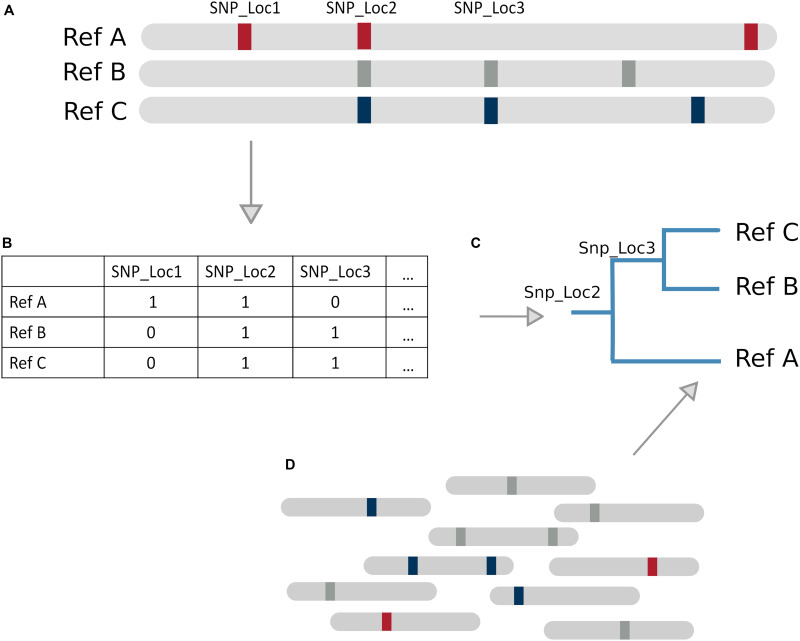
Tree Based Method Overview. **(A)** Example database of genomes with SNPs present as markers. **(B)** Representation of genome database, where 1 denotes a SNP and 0 absence of a SNP **(C)** SNP tree constructed based on SNPs from the database. **(D)** SNPs present in new reads can be matched against the tree to infer likely reference genome of origin by identifying sequences of successfully matching nodes (a path).

SNP based tree methods differ in their SNP calling, variant filtering, tree construction, and path determination techniques. Relying solely on SNPs limits the inclusion of other types of genomic variation such as indels, which could be picked up in a k-mer based method. SNP/phylogenetic hybrid methods are particularly suitable for species with low genomic divergence like *Mycobacterium tuberculosis*, because it is a clonal organism with strains differing by very few SNPs. Gan et al. (2016) and Sahl et al. (2015) (WG-FAST) have both developed tree based classification methods constructed using SNP variations between reference genomes (Figure 3). Another SNP based method, StrainEST (Albanese and Donati, 2017), is not based on a phylogenetic tree model but uses SNP frequencies within each genome of a reference database to predict strains based on co-occurring SNPs within a sample. This is done by modeling the SNP profile of a sample as a linear combination of the SNPs in a reference database using LASSO regression.

In contrast, k-mer based tree approaches can be more suitable for species that have larger degree of genetic variation or bigger structural variations that are not detectable by only considering SNPs. They would be less efficient at differentiating strains which are only a few SNPs apart as the impacts of a genetic sequencing error are more pronounced in the tree construction and classification process when working with k-mers. Roosaare et al. (2016) (StrainSeeker) have developed guide-tree based classification methods based on k-mers. A phylogenetic tree detailing the relationship between reference genomes must first be provided by the user.

Another kind of approach, GOTTCHA, generates a database of unique signatures for each genome at different taxonomic levels (Freitas et al., 2015). The unique signatures of a strain are the collection of all subsequences not found in any other available sequences at the desired taxonomic level. The unique signature of an unknown query sample can then be mapped against this database to determine coverage statistics for the query’s unique signature. The abundance of predicted strains is obtained through a statistic comparing the total number of mapped bases to the signature for the reference, and the number of unique bases mapped. StrainPhlAn (Truong et al., 2017) also uses species specific marker sets to classify strains, but only identifies the most abundant strain for each detected species in a metagenomic sample. The presence of other strains is assessed by calculating the number of polymorphic positions per species.

Other pattern based methods employ clustering to help delineate strains and augment pattern based detection techniques. For example, ConStrains assimilates elements of *de novo* assembly to detect genetically distinct strains (Luo et al., 2015). Reads for each species are first mapped against species-specific marker genes using MetaPhlAn2 (Segata et al., 2013) to generate a multiple alignment, and SNPs are determined using *Samtools* (Li, 2011) based on sufficient coverage criteria. The resulting SNP profiles are clustered into groups representing genetically distinct strains, with abundances calculated using a Monte-Carlo algorithm. In order to delineate strains, ConStrains requires a relatively high coverage (10×).

The major drawback of reference database methods (both pattern and alignment) is that detection of totally novel pathogens is not possible. In contrast, assembly based methods, which reconstruct genetically distinct genotypes without need for a reference, can detect and reconstruct novel strains. When confronted with a novel strain that is not represented in the reference database, a good reference database based detection method should output the nearest possible strain as well as the uncertainty of the match. Ultimately, meaningful results are limited to the identification of strains with reasonably close matches within the database.

### Reference Database Free Approaches

The methods described above all depend on either the presence of genome sequences in a reference database, or the reconstruction of a genome from reads. However, an additional subgroup of methods exist that do not use a reference database, but rather models within-sample diversity using a statistical model in order to delineate genetically distinct strains. These reference database free approaches apply statistics directly from elements acquired from the sequencing read set such as SNPs or k-mers.

For example, Eyre et al. (2013) applied a probabilistic model to allele frequencies at specific variable sites with the underlying assumption that the sample was a mixture of two haplotypes. Variable sites were defined across the whole genome as locations with ambiguous calls. As this approach is limited to modeling a maximum of two strains in the data, other methods have extended this approach to allow for the presence of multiple strains in the sample data, including *estMOI*, DEploid, and pfmix (Assefa et al., 2014; O’Brien et al., 2016; Zhu et al., 2017). Both DEploid and *estMOI* use variant calls to infer the number of haplotypes in the dataset first locally (short regions), then globally. DEploid goes further by using a reference panel of known genomes to create a prior in their Bayesian approach to estimate the relative abundance, number of haplotypes, and their allelic states. Pfmix similarly uses a Bayesian model but does not estimate haplotypes, instead uses a single reference to provide variants and allele frequencies to directly infer the number and proportions of strains from allele frequencies.

Reference database-free approaches do not attempt to identify the presence of a specific, previously sequenced strain; rather, they utilize allele (variant) discrepancies within a WGS read set to quantify the number and proportion of unique strains present in a sample. These methods are therefore unable to offer insight on the relationship of strains in the sample compared to previously documented strains, since there is no mapping of the sample to a database of previously seen strains. However, they are especially effective in determining strain number of species within cultured WGS samples.

## Comparative Discussion of Different Methodologies

**FIGURE 4:**
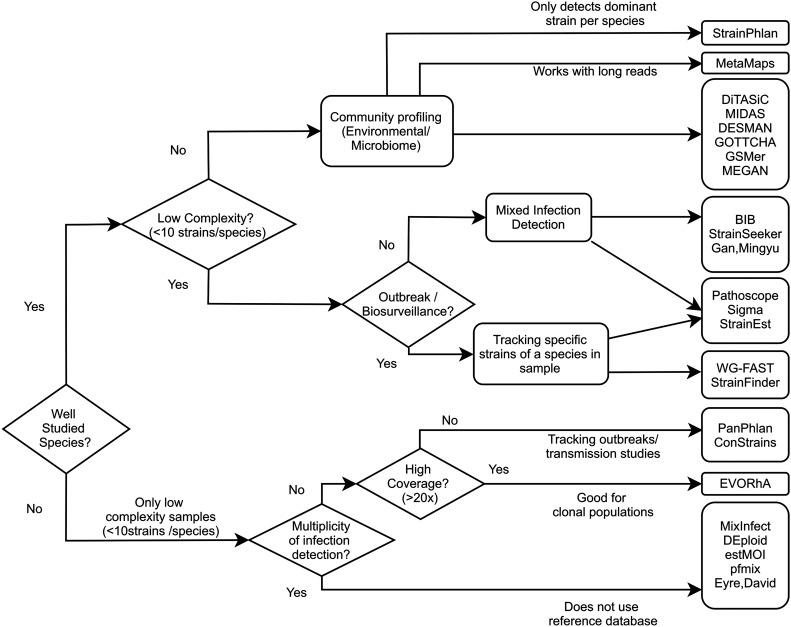
Flow chart of tool selection depending on scenario. Guide chart showing which tools can be used in which use case. Presence of a tool under one use case doesn’t necessarily exclude it from being applicable to another use case.

The methods mentioned in this review all aim to utilize the discriminative capability of WGS data to taxonomically classify samples at the level of individual strains within a species. These algorithms differ in required coverage, the number of strains that can be detected, the ability to detect higher level taxa (Table 2), and other criteria. To help guide tool selection we have made a flow chart (Figure 4) showing which types of tools would work well with different use cases.

**TABLE 2:** Tool use cases and detection details.

Method name	Taxonomic level^1^	A^2^	Sample setting^3^	Use cases^4^
EVORhA	strain	Y	– high coverage data	– reconstruct evolutionary trajectories – clonal populations – resolve genomes in metagenomic communities
DESMAN	strain	Y	– better with low complexity (<20 strains) communities	– environmental populations – metagenomic communities
Sigma	strain, species	Y	– made specifically to provide useful information for outbreaks	– metagenomic bio surveillance for outbreaks
BIB	strain	Y	– species with clear population structure and well-separated lineages – unsuitable for species with frequent recombination (maybe the case for many alignment methods)	– clinical use, mixed samples – flagging contaminated/problematic samples
Pathoscope	multiple levels	Y	– designed to be complete framework to analyze metagenomic data	– environmental samples – clinical samples
DiTASiC	strain	Y	– comparing abundances across samples	– general strain identification and abundance – allows for differential abundance testing across samples
MEGAN	strain, species	Y	– broad taxonomic classification	– environmental populations
MetaMaps	strain, species	Y	– long read data	– medium complexity environmental communities – medium complexity
StrainFinder	strain, species	Y	– track strain genotypes over time – specifically made to understand real world clinical problem – requires prior knowledge for number of strains	– clinical/pathogen identification – human microbiome
Gan, Mingyu	strain	Y	– specifically for TB	– clinical TB samples – mixed infections of few strains
ConStrains	strain, species	Y	– only needs one genome per species – robust against unknown strains	– clinical microbiome sets – time series data – finding specific strains within population at low abundance
GOTTCHA	user defined	Y	– designed to find low abundance populations	– clinical diagnosis – bio surveillance – community profiling
WG-FAST	strain	N	– isolate identification (single isolate and complex samples – designed for low coverage strains	– disease outbreaks – pathogen identification
StrainSeeker	strain, species	Y	– phylogeny based – identifying clade of novel strain – unable to differentiate strains with few SNV	– pathogen identification
StrainEst	strain	Y	– identifying strains of particular species – best at lower than species level – limited for poorly characterized species	– ecological/environmental samples – human/skin microbiome – molecular epidemiology
StrainPhlAn	strain, species	N	– identifies most abundant strain of particular species within metagenomes not all strains – reconstruction of stain level phylogenies of species	– human microbiome
MIDAS	strain, species	N	– cannot quantify novel species	– transmission gut microbiome
metaSNV	strain, species	N	– strain level variation within metagenomes	– environmental samples
GSMer	strain, species	Y	– identify species/strain specific for well-studied organisms – possible false negatives if not all GSMs covered – false positives due to overlapping GSMs with incorrect strains	– human microbiome
PanPhlAn	strain, species	Y	– characterization of strain level gene elements – useful for population genomics where few reference genomes exist – culture free	– outbreak epidemiology – human microbiome
MetaPalette	strain, speices	Y	– metagenomic profiling	– environmental samples – human microbiome
QuantTB	strain	Y	– specifically for TB	– mixed infections of few strains – clinical TB pathogen identification
Eyre, David W.	strain	Y	– mixed infection detection – assumes only mixes of 2 strains	– mixed infection screening in outbreak surveillance
pfmix	strain	Y	– mixed infection detection – specifically for *P. falciparum*	– pathogen identification
*estMOI*	strain	N	– specifically made for *P. falciparum* – estimates multiplicity of infection – might not be possible for highly related genomes	– pathogen identification – transmission intensity
DEploid	strain	Y	– estimating mixed infections – originally developed for *P. falciparum* – can be used for any mixture of strains within species	– pathogen identification
MixInfect	strain	Y	– detecting mixed infections in TB – not suitable for non-TB species	– pathogen identification

*^1^Taxonomy levels the method claims to be able to accurate identify. ^2^Denotes whether a method gives the abundance of a strain. ^3^Specifics about which context the tool was originally demonstrated for. ^4^Different use case scenarios that the tool can be used for or has been tested for.*

Reference database methods (alignment and pattern based) are the most broadly applicable group of methods. They can be used on samples with lower coverage levels of the species of interest (<1×) making them faster and more robust than assembly based approaches. In addition, they can be used to taxonomically classify or examine intra-species heterogeneity within an isolate culture expected to contain a single, well-studied species (such as *E. coli)*, as these methods require prior knowledge of a species. This is not possible for reference database free approaches. Also, some methods, such as GSMer and Sigma, are able to classify at both the species and strain level, which is useful when exploring strain level variety in metagenomic samples containing multiple species.

Biological uses of reference database methods can be quite broad. A common goal is to detect strains from only a particular pathogenic species. Pathoscope, SIGMA, WG-FAST, and PanPhlAN were all used to identify samples containing a particular toxic strain of *E. coli* from fecal metagenomic data obtained during a 2011 outbreak. In this case, although Sigma and Pathoscope are able to remove DNA from extraneous species, possibly providing a slight boost in computational efficiency, these methods are still both computationally intensive programs. Database methods can also be used to track transmission of strains between hosts. MIDAS was used to track strain transmission between mothers and their infants from stool metagenomes for a number of different microbial species. In a similar vein, StrainFinder was developed to track microbial strain transfer in fecal transplant cases. Phylogenetic-based methods such as those of Gan et al. (2016) and StrainSeeker can also track evolutionary divergence of the same strain within longitudinal metagenomic samples. These methods have the advantage of including a visual representation of the underlying decision process which can be easier to explain and understand. The phylogenetic framework also offers users the ability to sanity check results. For example, multiple closely related strains can be detected when the “true” strain is not present in the database.

If the single species present in the isolate sample is not as well-studied, then assembly methods are suitable, as they are not as dependent on prior knowledge encapsulated in a reference database. Assembly methods can also be useful in tracking progression of a single genome. For example, EVORhA was used to examine an evolving clonal population of *E. coli* strain. Because assembly methods require sufficient coverage (50 × for EVORhA) to resolve haplotypes, these methods are not suitable for communities of samples with low coverages.

Certain methods quantify the number of strains or the relative abundance of strains within a sample using allelic variations within the dataset and do not require a database of known genomes. These reference database free tools are useful when the relationships between strains in a single-species sample are of interest, rather than the exact strain identities or their relationships to previously studied strains. This would be suitable for testing multiplicities of strains in uncultured soil samples or other extreme environments which are still under sampled. Reference database free approaches can also be applied for well characterized species, however, since pattern and alignment based tools can also offer strain identity – these might be preferred due to the extra information given.

Ease of use and speed of analysis are both important concerns when considering a metagenomic tool. Table 1 details the different machine requirements and speed tests given by the methods reported in this paper. Though versatile and adaptable to different scenarios, tools requiring extensive mapping to a reference database can be extremely computationally intensive. Sigma required nearly 20 h resolving a single 5 strain community (20 million reads) against a database of 2,266 reference genomes with 62GB of memory and 64 cores. StrainFinder, another alignment method, took more than 48 h with 100–200 cores for 100 samples. Some methods were tested in high performance computing environments (i.e., Pathoscope, MEGAN, GOTTCHA, all >100GB memory) which may not always be available for clinicians. Additionally, tools requiring a database typically only report times/requirements to process a sample, but rarely include the time required to generate a custom database. We were only able to find both values for StrainSeeker, which process samples relatively quickly (<2 min) but suggests 300GB of space and 512GB of ram available to generate a database. In terms of usability, almost all of the tools were made to run in a Linux environment, therefore requiring some level of computational expertise in order to navigate requirements and installation setups. Few tools offer an online accessible functionality (MEGAN and StrainSeeker). That being said, certain tools are bundled in easy to install package managers like Conda and R (i.e., DEploid, pfmix, StrainPhlAn), while others only offer a collection of scripts (i.e., MixInfect, and Eyre et al). Due to the requirements for installation and use (Bash/Linux), using most of these methods would require some bioinformatics knowledge. Further work would need to go into making these tools accessible and open for general use, such as online web tools, or a easy to use/install gui.

Most of the methods described in this review have not been benchmarked across all possible use case scenarios in a systematic or independent manner; therefore, a researcher using these tools will need to carefully determine whether a particular tool would work for their data type of interest. We discuss more about benchmarking in the next section.

## Method Evaluation, Benchmarking and Simulation

Thorough and robust benchmarking of algorithms for a particular application and data type is critical. As this field is relatively new, there has yet to be a proper comparative study benchmarking the efficiency, accuracy, and specificity of these methods in a diversity of application domains: clinical pathogens (Cassir et al., 2016; Ward et al., 2016), microbiomes (Fang et al., 2018; Goltsman et al., 2018) and industrial biotechnology (Capece et al., 2016; Walsh et al., 2017; De Filippis et al., 2019) as examples.

The types of validation that have been performed for each method are indicated in Table 1. For all tools, an initial validation of model performance was performed using *in silico* simulated reads of known composition, generated from genomes of known host strains using tools such as *MetaSim, Grinder*, and *Art* (Richter et al., 2011; Angly et al., 2012; Huang et al., 2012). Alternatively, sequencing reads from presumed pure strains can be used. Testing applicability to strain mixes involves constructing a more complex synthetic dataset containing a mixture of varying quantities of individual strain read sets. Factors that must be considered in the construction of synthetic validation datasets include: (1) Determining the actual sequencing depth necessary to be able to identify a particular strain in a read set and number of reads to use. (2) The diversity in strain composition in terms of taxonomic levels that should be represented or background non-target species. (3) The level of complexity that needs to be introduced in the reads (in terms of SNVs and genomic distance between strains) and (4) the scalability of the method to fluctuation in sample size (e.g., low abundance strains in large sample sets). Validation on synthetic datasets addresses performance of the algorithms in the best-case scenario. Subsequent to these validation experiments, performance needs to be examined on test-case “real” samples, as this is often presents a much greater challenge than testing on *in silico*-generated datasets.

In order to compare the results of benchmarking different tools, metrics for comparing results across different types of outputs from various tools must be carefully chosen. The published benchmarking methods for the tools described in Table 1 use a variety of different metrics. The most common method employed for the published tools involves testing the specific algorithm on a dataset of known diversity and abundance, and comparing accuracy metrics. For alignment- and pattern based methods, a true and false positive would be defined as whether the algorithm was able to detect the correct strain within the sample, or whether it detected the wrong strain, respectively. A false negative would be defined if the algorithm failed to detect a strain present in the sample, and a true negative would be called if the algorithm did not output any strains not present. An important consideration in the assessment of true negatives is whether the algorithm informs the user of the uncertainty of the match and outputs the nearest strain. Most methods mentioned in this paper quantified the reliability of their method by either calculating the true positive rate/false discovery rate or by checking manually whether the results were correct.

In addition to simply identifying which strains are present or absent in a sample, additional metrics must assess the accuracy in estimating strain abundances. One method to do this, used by the assembly based detection method, EVORhA, uses the mean absolute error (MAE) metric between the true abundances and estimated abundances. In addition, they also calculated the root mean squared error (RMSE), which was also used by Eyre et al. Another method to assess accuracy in strain abundance is the Jenson-Shannon divergence, which was used in ConStrains to measure their prediction accuracy.

A comprehensive comparison and benchmarking of these tools is needed to provide further insight into the efficiency of these tools at performing strain-level identification on a wide range of sample types, be it metagenomic, clinical, or cultures. This benchmarking strategy would need to deal with the nuances between tools, as they have different goals, different use-case scenarios, and different criteria for success. It might be possible to conduct these comprehensive benchmarks in categories such that similar tools could be evaluated together on novel datasets with a common evaluation metric.

## Conclusion and Future Directions

Whole genome sequencing of microbial populations has the capability to offer a view into genetic diversity at varying taxonomic levels. Current widely used taxonomic classifiers allow for the identification of species within WGS sets. However, algorithms for finer-grained classification, at the individual strain level within a species, are still relatively new. Such techniques have the capacity to greatly impact healthcare and other fields by precise tracking of disease outbreaks, differentiation of commensal and pathogenic strains, and linking strain level genotypic traits with phenotypic characteristics of clinical and industrial importance (Capece et al., 2016; Cassir et al., 2016; Ward et al., 2016; Walsh et al., 2017; Fang et al., 2018; Goltsman et al., 2018; De Filippis et al., 2019). One assumption almost universally made within taxonomic tools is that a direct relationship exists between strain read coverage and strain abundance in the sample. As such, calculations of strain abundance levels take into account the variations of coverage across variant sites or reads. Though intuitive, none of the tools presented here presented analysis to prove this assumption. Conducting such verification steps is particularly important for tools focusing on clinical use and pathogen identification, where it is typical for a culturing step to be conducted before sequencing. In actuality, there could be many reasons why read abundance does not directly reflect the composition of the sample: isolation technique (culture sweep vs. single colony isolation), cell lysis efficiency, contamination skewing read depth, or the sequencing process itself (Morgan et al., 2010; Pereira et al., 2018).

There are numerous ways in which current strain identification methods can improve their benchmarking. Firstly, very few algorithms tested the performance of their tools on multiple (>2) low abundance strains (<1–2×). Detecting low abundance strains would be preferred for microbial communities such as the gut, where specific strains exhibit differing pathogenicity. Secondly, no methods quantified or benchmarked how genetically distant a strain needs to be in order to properly delineate it. Third, there are no tools that allow a user to compare strains within and across samples, which would be useful for transmission studies. Lastly, delineating extremely closely related strains remains a difficult problem for the metagenomic tools. Many tools requiring a reference database remove genomes from the database that are extremely close together or self-report that they would not work well with highly related genomes (Assefa et al., 2014; Sankar et al., 2015; Albanese and Donati, 2017). Such analysis remains difficult due to the problems that arise when considering closely related strains such as an increase in false positives due to both strains being reported when only one is actually present or problems within the model itself driven by high levels of collinearity. The difficulty with detecting extremely close strains is further compounded due to the ambiguous definition of a strain.

The methods detailed in this literature review are almost all directed toward sequencing technologies that produce reads from mixtures of cells. Direct sequencing of individual cells would bypass this need to computationally subdivide reads produced from current NGS technologies into those originating from different strains. Single-cell sequencing strategies such as Drop-Seq (Macosko et al., 2015) and 10× Genomics (Zheng et al., 2017) are rapidly improving to provide a systematic and comprehensive view of the genetic diversity of complex communities. Having sequencing data originating from individual cells would greatly simplify studies of heterogeneous populations of strains. However, there are still technical difficulties to overcome before single-cell sequencing becomes widely adopted. It is probable that the next iteration of strain-level identification algorithms will be focused on such technologies. One pioneering example is *MetaSort*, which combines the advantages of both WGS and single cell sequencing data (Ji et al., 2017). This method assembles genomes from both WGS reads and single cell sequencing reads and integrates the two using a machine-learning algorithm, resulting in genomes present in the sample. The increased resolution from single cell sequencing based detection is likely to uncover novel forms of genetic heterogeneity. In addition, advances in long read sequencing continue to change the scope and direction of strain-level detection in metagenomic samples.

Longer read lengths could make it easier and more practical to phase haplotypes, as well as identify strains with fewer reads. A number of studies have applied long read sequencing data from third generation sequencing platforms such as Pacific Biosciences (PacBio) and Oxford Nanopore Technologies (ONT) to assemble individual strains within metagenomic communities (Tsai et al., 2016; Bertrand et al., 2019). For example, Somerville et al. (2019) used the long-read assembler, Flye (Kolmogorov et al., 2019), to reconstruct individual contigs from a long read metagenomic sample, followed by a phylogenetic analysis using NCBI RefSeq to determine strain identity. Long-reads can also be beneficial for alignment based strain identification approaches. For example, MetaMaps developed its own mapping algorithm to align long reads to genomes in a database. Challenges for strain-level identification using long-read sequencing can vary based on the tools. In the case of MetaMaps, a minimum read-length is required for a read to be considered, resulting in numerous unassigned reads. Overall, the use of longer reads can mitigate some of the limitations of short-reads, allowing for the resolution of difficult to sequence regions and longer contigs. However, this comes at the expense of increased errors, lower coverage and higher cost. We still expect many more tools will be released for long-read platforms as it continues to gain in popularity.

The ability to quantify and detect bacterial strains within heterogeneous environments has applications in numerous fields including diagnostics (Dekkera, 2018), clinical studies for the microbiome (Wang et al., 2015), bio surveillance (Ahn et al., 2015), tracking transmission of infectious strains in an outbreak (Hong et al., 2014; Ahn et al., 2015; Nayfach et al., 2016), providing insight into the spread of antibiotic resistance (Sukhum et al., 2019), tracking progression of within-host bacterial evolution (Pulido-Tamayo et al., 2015) and exploring diverse environments (Tringe and Rubin, 2005). We look forward to the wide range of applications and effects these tools will have in shaping and progressing sequencing based research.

## Author Contributions

CA and TA conceived, designed, and wrote the manuscript. AM, TS, and AE edited and proofread the manuscript. All authors contributed to the article and approved the submitted version.

## Conflict of Interest

The authors declare that the research was conducted in the absence of any commercial or financial relationships that could be construed as a potential conflict of interest.
